# Social Pathology and Education

**Published:** 1894-04-07

**Authors:** 


### Studies in Applied Sociology.

### VI.—SOCIAL PATHOLOGY AND EDUCATION.

What can we do for the child of the slum—the diseased product of our complicated civilisation? The healthy products find their place and function, not always with ease, but always in the end; but for these dwarfed, ill-balanced pathological specimens, what can we do to make them worthy of a place at all? Some years ago a certain illustrated advertisement was very common. At one end of the poster was a child's head, graceful and innocent, if somewhat inane, with, subjoined, the question, "What will he become?" From it branched two series of portraits, the good and the bad, culminating above in "honoured age," below in "beggary." It was the advertisement of a "popular educator," and the implication was that in education, moral and social, salvation lay.

This is true; but before assuming that we have here a remedy for all the ills that soul is heir to we must ask two questions, "Will the child accept education?" and "What education is most suited for him?" Applying the first question to the child of the slum, the answer is brief and conclusive: "No, not if he can help it." He does not desire education; neither do his parents desire it for him. They themselves will teach him all they think it needful he should know—dodges and ingenuities which with only occasional deviations into work or crime will keep him, like themselves, a member of the "leisured class among the poor"; and they aid and sympathise with his efforts to evade the tyranny of the School Board. The result is that the slum child, the offspring of the casual poor, costs more to educate than the child of the well-to-do artisan, and knows next to nothing at the end. The actual figures are £3 6s. 3d. as against £2 14s. 4d. per head per annum. These more expensive children belong to the districts where starvation reigns; a sufficiency of either food or clothing is unknown to them, though for their parents' comfort there exist in their neighbourhood public-houses in the proportion of 1 to every 100 adults.

Being what they are, what education is the best for them? It may be admitted that no one is much the worse for knowing the three R's; but beyond this it may be doubted if the School Board really does much for the child of the slum, or even for children higher in the social scale. Compulsory education is not thrust upon people by way of bestowing on them an intellectual luxury, but to fit them to bear their part in the battle of life. Thus looked at, a great part of our educational code is unpractical. It is felt to be so by parents of even the best working class, who therefore take their children away from school as soon as the law allows, and make them errand boys for the sake of the two or three shillings a week they can add to the family income; though they would sacrifice the present advantage if they felt that the education their children are receiving would help them in after life. If parents with a true sense of their responsibilities feel this, it cannot be wondered at that our educational system fails to develop the parental instincts in the tramp and the casual worker. At the best these people are children themselves, living from hand to mouth, heedless either of their own future or their children's, and they see nothing in education to meet their approval, and give them the notion of setting present loss against future gain.

It is impossible, of course, to make the school the workshop of every trade; but surely, considering the future destiny of these children, more prominence should be given to manual training. A man who can use his hands need neither starve nor steal, even in an old country. True, we hear every winter a clamour about the unemployed; but in most cases it will be found that the unemployed are those who cannot work—who know no trade thoroughly, and are taken on only for the most mechanical jobs when work is particularly plentiful. Good workmen of sober habits do not bulk largely in the crowds who stand idle in the market place. It is possible to teach the use of three carpenters' tools with one piece of wood six inches long, and instruction of this kind is more attractive as well as more useful to the average boy than a knowledge of Cæsar's Gallic wars. Though all are not to be carpenters, all may get from this one simple exercise training for eye and hand, which will never come amiss in any trade. Gymnastic training and drill are valuable too, though subordinate in some respects to manual training; but so many children of the poorest class are so unfit physically to hold their own that an essential part of their education is to give them as much bodily strength as possible. This need forms also an excuse for giving children free meals at school, though we fear that on the score of political economy one can hardly approve of charities which tend to lessen the responsibilities of parenthood. Free dinners to the children mean too often the contented acceptance of lower wages by the father.

With regard to girls, the course of true education is simple enough, and is being largely followed. Cookery classes in schools are becoming common, and are distinctly popular with the pupils. Laundry work also is being introduced, and sewing, though with impractical elaboration of tucking and feather-stitching, is carefully taught. Every feminine heart takes naturally to these womanly accomplishments, and though many of the girls may not have in their own homes the material for turning their knowledge to account, they acquire principles and habits of work which will teach them how to make the best use of the materials to their hand, and to use others profitably when opportunity occurs. The girl of the slums as she now comes forth from school is not fit for even the meanest sort of domestic service, and domestic service, besides being on the whole the safest and most comfortable life for working girls, is undeniably the best training for the destiny to which they all aspire. Mr. Besant says in a recent novel that nobody knows how ignorant a human creature can be till he has married a factory hand. That it should be so is a reproach not only to the girls, but to the schools where they are taught. We would beg educators and educational legislators to remember that it is not enough to develop the brain if we do not also teach the brain to inform the hand.

